# Characterization of a male reproductive transcriptome for *Peromyscus eremicus* (Cactus mouse)

**DOI:** 10.7717/peerj.2617

**Published:** 2016-10-27

**Authors:** Lauren L. Kordonowy, Matthew D. MacManes

**Affiliations:** Department of Molecular, Cellular, and Biological Sciences, University of New Hampshire, Durham, NH, United States

**Keywords:** *Peromyscus eremicus*, Transcriptome, Genomics, Bioinformatics, Adaptation, Desert physiology, Cactus mouse, Reproduction

## Abstract

Rodents of the genus *Peromyscus* have become increasingly utilized models for investigations into adaptive biology. This genus is particularly powerful for research linking genetics with adaptive physiology or behaviors, and recent research has capitalized on the unique opportunities afforded by the ecological diversity of these rodents. Well characterized genomic and transcriptomic data is intrinsic to explorations of the genetic architecture responsible for ecological adaptations. Therefore, this study characterizes the transcriptome of three male reproductive tissues (testes, epididymis and vas deferens) of *Peromyscus eremicus* (Cactus mouse), a desert specialist. The transcriptome assembly process was optimized in order to produce a high quality and substantially complete annotated transcriptome. This composite transcriptome was generated to characterize the expressed transcripts in the male reproductive tract of *P. eremicus,* which will serve as a crucial resource for future research investigating our hypothesis that the male Cactus mouse possesses an adaptive reproductive phenotype to mitigate water-loss from ejaculate. This study reports genes under positive selection in the male Cactus mouse reproductive transcriptome relative to transcriptomes from *Peromyscus maniculatus* (deer mouse) and *Mus musculus.* Thus, this study expands upon existing genetic research in this species, and we provide a high quality transcriptome to enable further explorations of our proposed hypothesis for male Cactus mouse reproductive adaptations to minimize seminal fluid loss.

## Introduction

The rapid infusion of novel bioinformatics approaches in the fields of genomics and transcriptomics has enabled the coalescence of the fields of genetics, physiology and ecology into innovative studies for adaptation in evolutionary biology. Indeed, studies on the biology of adaptation had previously been dominated by research painstakingly documenting morphological shifts associated with ecological gradients (e.g., in *Peromyscus*: Carleton, 1989; MacMillen & Garland, 1989). However, the discipline of bioinformatics has breathed new life into the field of adaptation biology. Specifically, while the morphological basis as well as the physiological mechanisms of adaptation have been explored for a variety of species in extreme environments, the genetic underpinnings of these adaptations have only recently become a larger area of research (Hoekstra et al., 2006; Cheviron & Brumfield, 2011; Lorenzo et al., 2014; Guillen et al., 2015). High-throughput sequencing technology of model and non-model organisms (Ellegren, 2014) enables evolutionary biologists to conduct genome and transcriptome wide analyses and link patterns of gene selection with functional adaptations.

Studies on the genetic basis of adaptation have included a wide variety of taxa. For example, butterflies in the *Heliconius* genus have been a particularly effective study system for determining the genetic basis of pigmentation patterns, and there is evidence of interspecific introgression for genes enabling adaptive mimicry patterns (Hines et al., 2012; *Heliconius* Genome Consortium, 2012). In addition, a population genomic study in three-spine sticklebacks has elucidated many loci responsible for divergent adaptations from marine to freshwater environments (Jones et al., 2012). Furthermore, researchers have developed a list of candidate genes that have evolved in multiple populations of freshwater adapted three-spine sticklebacks (Hohenlohe et al., 2010). Another active area of adaptation genetics research focuses on species residing in extreme environments. High altitude adaptations to hemoglobin variants have been identified in multiple organisms, including humans (Lorenzo et al., 2014), several species of Andean ducks (McCracken et al., 2009a; McCracken et al., 2009b), and deer mice, *Peromyscus maniculatus* (Storz et al., 2010; Natarajan et al., 2015). The genetic pathways responsible for physiological adaptations to desert habitats remain enigmatic; however, considerable progress has been made developing candidate gene sets for future analyses (e.g., Guillen et al., 2015; MacManes & Eisen, 2014; Marra et al., 2012; Marra, Romero & DeWoody, 2014). Functional studies will stem from this foundational research aimed at identifying the genomic underpinnings of adaptations to extreme environments; yet, it is inherently challenging and critically important to demonstrate that specific loci are functionally responsible for adaptations (Storz & Wheat, 2010).

Rodents of the genus *Peromyscus* have been at the forefront of research elucidating the genetic basis for adaptation (reviewed in Bedford & Hoekstra, 2015). This diverse genus has served as an ideal platform for adaptation research spanning from the genetic basis of behavioral adaptations—such as complex burrowing in *Peromyscus polionotus* (Weber & Hoekstra, 2009; Weber, Peterson & Hoekstra, 2013)—to the loci responsible for adaptive morphology—such as coat coloration in *Peromyscus polionotus leucocephalus* (Hoekstra et al., 2006), and including kidney desert adaptations in *Peromyscus eremicus* (MacManes & Eisen, 2014). We are currently using *Peromyscus eremicus* as a model species for investigating the genetic bases of desert adaptations. This paper describes a crucial component of this research aim.

Initial steps toward understanding the genetics of adaptation must include the genomic and transcriptomic characterization of target study species (MacManes & Eisen, 2014). To this end, we assembled and characterized a composite transcriptome for three male reproductive tissues in the desert specialist, *P. eremicus*. This species is an exceptional example of desert adaptation, as individuals may live entirely without water access (Veal & Caire, 2001). MacManes & Eisen (2014) assembled transcriptomes from kidney, hypothalamus, liver, and testes of this species, and they identified several candidate genes potentially underlying adaptive renal physiology. However, to our knowledge, potential physiological reproductive adaptations to water limitation have not been studied in this species or in other desert rodents. We hypothesize that male *P. eremicus* possess reproductive adaptions to mitigate water loss.

The adaptive kidney physiology in kangaroo rat species (genus: *Dipodomys*), which produce highly concentrated urine via a disproportionately long loop of Henle (Schmidt-Nielsen et al., 1948; Schmidt-Nielsen & Schmidt-Nielsen, 1952; Vimtrup & Schmidt-Nielsen, 1952; Urity et al., 2012), has been well-established. We propose that there may also be reproductive adaptations to mitigate water loss in desert rodents. Recent findings pertaining to the genetic signatures of adaptive kidney function in desert rodents (Marra et al., 2012; Marra, Romero & DeWoody, 2014; MacManes & Eisen, 2014), suggest that such hypothesized reproductive adaptations may be detectable at the genetic level (should they exist) using similar comparative transcriptomic methods. We present this hypothesis in light of the mounting body of research for high rates of reproductive protein evolution (reviewed in Swanson & Vacquier, 2002; Ramm et al., 2014), which we propose indicates that reproductive tissues may possess a significant capacity for evolving in response to strong selective pressures.

Dewsbury (1982) made the assertion that producing ejaculates incurs a cost for male mammals, which produce relatively high sperm counts, and his analysis utilizes *P. maniculatus* as a model. Ejaculation has also been demonstrated to be costly in *Mus musculus domesticus*; Ramm & Stockley (2007) found that mice are able to manipulate the quantity of sperm released in response to varying competition for females. *Peromyscus* and other rodents exhibit rapid evolution of testis-expressed proteins (Turner, Chuong & Hoekstra, 2008). In addition, the epididymal transcriptome of *M. musculus* shows evidence for positive selection among epididymis-specialized genes that are secreted, which the authors attribute to their putative evolutionary importance (Dean, Good & Nachman, 2008). Moreover, a recent analysis for sperm genes from multiple mouse strains found that sperm proteins involved in both motility and in sperm-egg interactions show signatures of positive selection, potentially facilitating evolutionary mechanisms for sperm-competition and sexual conflict (Vicens, Luke & Roldan, 2014). We therefore propose that both the inherently costly nature of producing ejaculate and the rapid evolution of genes in murine testes, epididymis, and sperm would be ideal for desert rodents to evolve ejaculate adaptations to limited water-availability.

In order to develop genomic resources that will allow us to begin to test our hypothesis related to reproductive water conservation, we developed a transcriptome comprising three male specific reproductive tissues in *P. eremicus*. Specifically, we assembled a composite reproductive transcriptome for the epididymis, testes, and vas deferens. These tissues were chosen because they constitute numerous physiological roles in spermatogenesis and in the generation and transportation of seminal fluids. We posit that one or more of these reproductive tissues possess phenotypic characteristics to mitigate water loss from seminal fluids. We also propose that any molecular mechanisms responsible for such an adaption should be elucidated through future differential gene expression and comparative transcriptomic studies. However, the initial assembly and characterization of a comprehensive male reproductive tissue transcriptome in the current manuscript will be essential for research studies exploring this hypothesized male *P. eremicus* reproductive desert adaptation. For example, this transcriptome will be instrumental in further experimental studies investigating differential gene expression in these tissue types in response to variable hydration levels or in large-scale comparative transcriptomic studies spanning numerous desert and non-desert rodents.

Here, we characterize a male Cactus mouse tissue-specific transcriptome by presenting a preliminary exploration of transcript abundance and putative homology. We also perform comparative transcriptomic analyses to identify evidence of positive selection in genes potentially related to the hypothesized reproductive desert adaptations. It is beyond the scope of this manuscript to evaluate the functionality of these candidate genes in the context of desert adaptation, much less male specific reproductive adaptations. However, the elucidation of candidate genes in the context of male reproductive tissues will be instrumental for future studies aimed at determining which genes are functionally responsible for the proposed reproductive adaptations to water limitation in male Cactus mouse.

## Methods

### Tissue samples, RNA extraction, cDNA library preparation and sequencing

The *Peromyscus eremicus* male used for this study was captive born and descendant from a population from the *Peromyscus* Genetic Stock Center (Columbia, South Carolina). This individual was housed in a facility at the University of New Hampshire designed to mimic desert conditions in the Southwestern United States. Specifically, the temperature increases gradually during the light hours until it peaks at 90°Fahrenheit in the afternoon, and the temperature decreases during hours of darkness to 75°. Humidity levels are 10% during the daylight hours and 25% during darkness. The photoperiodic cycle in this desert chamber is for long days of photo-stimulation, with 16 h of light, and 8 h of darkness. The colony includes males and females which are housed within a single room, providing olfactory cues that stimulate reproductive maturity. The photoperiod and shared housing in this colony result in the attainment of reproductive maturity in both sexes. Males are deemed reproductively mature when they are fully scrotal. The males do not undergo seasonal testicular atrophy, as evidenced by their consistent scrotal condition and their year-round successful reproduction.

A single reproductively mature *P. eremicus* male was sacrificed via isoflurane overdose and decapitation. This was done in accordance with University of New Hampshire Animal Care and Use Committee guidelines (protocol number 130902) and guidelines established by the American Society of Mammalogists (Sikes et al., 2011). Testes, epididymis, and vas deferens were immediately harvested (within ten minutes of euthanasia), placed in RNAlater (Ambion Life Technologies) and stored at −80 °C until RNA extraction. We used a standard TRIzol, chloroform protocol for total RNA extraction (Ambion Life Technologies). We evaluated the quantity and quality of the RNA product with a Qubit 2.0 Fluorometer (Invitrogen) and a Tapestation 2200 (Agilent Technologies, Palo Alto, USA).

We used a TURBO DNAse kit (Ambion) to eliminate DNA from the samples prior to the library preparation. Libraries were made with a TruSeq Stranded mRNA Sample Prep LS Kit (Illumina). Each of the three samples was labeled with a unique hexamer adapter for identification after multiplex single lane sequencing. Following library completion, we confirmed the quality and quantity of the DNA product with the Qubit and Tapestation. We submitted the multiplexed sample of the libraries for running on a single lane at the New York Genome Center Sequencing Facility (NY, New York). Paired end sequencing reads of length 125 bp were generated on an Illumina 2500 platform. Reads were parsed by tissue type according to their unique hexamer IDs in preparation for transcriptome assembly.

### Reproductive transcriptome assembly

The composite reproductive transcriptome was assembled with reads from the testes, epididymis and vas deferens using the previously developed Oyster River Protocol for *de novo* transcriptome assembly pipeline (MacManes, 2016). Briefly, the reads were error corrected with Rcorrector v1.0.1 (Song & Florea, 2015). We used the *de novo* transcriptome assembler Trinity v2.1.1 (Haas et al., 2013; Grabherr et al., 2011). Within the Trinity platform, we ran Trimmomatic (Bolger, Lohse & Usadel, 2014) to remove the adapters, and we also trimmed at PHRED < 2, as recommended by MacManes (2014).

Next we evaluated transcriptome assembly quality and completeness using BUSCO v1.1b1 and Transrate v1.0.1. BUSCO (Simão et al., 2015) reports the number of complete, fragmented, and missing orthologs in assembled genomes, transcriptomes, or gene sets relative to compiled ortholog databases. We ran BUSCO on the assembly using the ortholog database for vertebrates, which includes 3,023 genes. The assembly was also analyzed by Transrate using the *Mus musculus* peptide database from Ensembl (downloaded 2/24/16) as a reference. The Transrate score provided a metric of *de novo* transcriptome assembly quality, and the software also generated an improved assembly comprised of highly supported contigs (Smith-Unna et al., 2016). Finally, we re-ran BUSCO on the improved assembly generated by Transrate to determine if this assembly had similar metric scores for completeness as the original assembly produced by Trinity. As alternatives to the original Trinity assembly and the optimized Transrate assembly, we proceeded with our optimization determinations by filtering out low abundance contigs from the original Trinity assembly. First we calculated the relative abundance of the transcripts with Kallisto v0.42.4 and Salmon v0.5.1. Kallisto utilizes a pseudo-alignment algorithm to map RNA-seq data reads to targets for transcript abundance quantification (Bray et al., 2015). In contrast, Salmon employs a lightweight quasi-alignment method and a high speed streaming algorithm to quantify transcripts (Patro, Duggal & Kingsford, 2015). After determining transcript abundance in both Kallisto and Salmon, we removed contigs with transcripts per million (TPM) estimates of less than 0.5 and of less than 1.0 in two separate optimization trials (as per MacManes, 2016). Finally, we evaluated these two filtered assemblies with Transrate and BUSCO to determine the relative quality and completeness of both assemblies. We chose the optimal assembly version by comparing Transrate and BUSCO metrics and also through careful consideration of total contig numbers across all filtering and optimizing versions. The chosen assembly was the Transrate optimized TPM > 0.5 filtered assembly, and this assembly was used for all subsequent analyses.

### Annotation, transcript abundance, and database searches

We used dammit v0.2.7.1 (Scott, 2016) to annotate the optimized transcriptome assembly (as per MacManes, 2016). Within the dammit platform, we predicted protein coding regions for each tissue with TransDecoder v2.0.1 (Haas et al., 2013), which was used to find open reading frames (ORFs). Furthermore, dammit utilizes multiple database searches for annotating transcriptomes. These database searches include searches in Rfam v12.0 to find non-coding RNAs (Nawrocki et al., 2015), searches for protein domains in Pfam-A v29.0 (Sonnhammer, Eddy & Durbin, 1997; Finn et al., 2016), the execution of a LAST search for known proteins in the UniRef90 database (Suzek et al., 2007; Suzek et al., 2015), ortholog matches in the BUSCO database, and orthology searches in OrthoDB (Kriventseva et al., 2015).

We used the assembly annotated by dammit to re-run Kallisto to determine transcript abundance within each of the three tissue types. We used TPM counts of expression for all three tissues to generate counts of transcripts specific to and shared across tissue types. We also downloaded the ncRNA database for *Mus musculus* from Ensembl (v 2/25/16), and we did a BLASTn (Altschul et al., 1990; Madden, 2002) search for these ncRNAs in our assembly. This database has 16,274 sequences, and we determined the number of transcript ID matches and the number of unique ncRNA sequence matches for our assembly. We also counted how many transcript matches were present in each of the tissues, and we referenced the corresponding Kallisto derived TPM values to determine the number of unique and ubiquitous transcript matches for each tissue.

We searched the annotated assembly for transporter protein matches within the Transporter Classification Database (tcdb.org). This database has 13,846 sequences representing proteins in transmembrane molecular transport systems (Saier et al., 2014). We executed a BLASTx (Altschul et al., 1990; Madden, 2002) search to find the number of transcript ID matches and the number of unique transporter protein matches within the assembly. Next we determined how many transcript ID matches were found in each of the three tissues. As previously described above, we also cross-referenced these matches with the Kallisto derived TPM values to find the number of ubiquitous and unique transcript matches by tissue type.

### Comparative analysis for genes under positive selection

We performed a three-species comparative analysis to identify genes under positive selection in the male reproductive transcriptome for the *P. eremicus* lineage relative to *M. musculus* and *P. maniculatus*. The *M. musculus* nucleotide and protein sequences were downloaded (version GRCm38) from Ensembl (ensembl.org). The *P. maniculatus* nucleotide and protein files were downloaded (version GCF_000500345.1_Pman_1.9) from NCBI (ncbi.nlm.nih.gov). The comparative analysis was conducted as described below. The corresponding nucleotide and protein files for all three species were modified so that the header names for the sequences in each species file pair were concordant. Next, we found orthologous groups of protein sequences using OrthoFinder v0.6.1 (Emms & Kelly, 2015). The two OrthoFinder command scripts aligned sequences with MAFFT v7.123b (Katoh et al., 2002), built trees with FastTreeMP v1 (Price, Dehal & Arkin, 2010), and generated orthogroups based on sequence similarity. Our next script selected the single copy orthologs (SCOs) from among these orthogroups for analysis. Then we selected the cds file transcripts for all three species corresponding to the previously identified single copy orthologs. Finally, we ran a script which aligned the sequences with PRANK v150803 (Löytynoja, 2014) and performed the analyses for positive selection for the single copy orthologous sequences with codeml in PAML v4.8 (Yang, 1997; Yang, 2007). Specifically, we performed the *M*2*a* branch site test (Zhang, Nielsen & Yang, 2005) for positive selection after stipulating *P. eremicus* as the foreground lineage. Genes were deemed under positive selection if the omega values (w) exceeded 1 within the *M*2*a* model and if they yielded statistically significant results for the likelihood ratio test (LRT) comparison between the null and alternative models. To perform the LRT, we determined if 2ΔlnL (two times the difference between the log likelihood values for the alternative—*M*2*a*—and the null—*M*1*a*—models) exceeded a chi-square value corresponding to a significant *p*-value (*p* < 0.05) (Yang, 1998) after applying the Benjamini-Hochburg correction for multiple comparisons (Benjamini & Hochberg, 1995). For each of the *P. eremicus* gene sequences demonstrated to be under positive selection according to the LRT, we selected the *M. musculus* cds sequence from the SCO group corresponding to the *P. eremicus* gene sequence. Then we performed a BLASTn search on the *M. musculus* sequence to find gene matches on NCBI to determine the gene identity for the *P. eremicus* sequence under positive selection.

Of note, the code for performing all of the above analyses can be found at GitHub (https://github.com/macmanes-lab/peer_reproductive/transcriptome). The data files are available on Dryad (doi: 10.5061/dryad.01c3t).

## Results and Discussion

### Reproductive transcriptome assembly

There were 45–94 million paired reads produced for each of the three transcriptome datasets, yielding a total of 415,960,428 reads. The raw reads are available at the European Nucleotide Archive under study accession number PRJEB13364.

We assembled a *de novo* composite reproductive transcriptome with reads from testes, epididymis and vas deferens. The evaluation of alternative optimized assemblies allowed us to generate a substantially complete transcriptome of high quality. The alternative assemblies had raw Transrate scores ranging from 0.156–0.194 (Table 1). However, the scores for the improved assemblies generated by Transrate, consisting of only highly supported contigs, ranged between 0.285 and 0.349, which is well above the threshold Transrate score of 0.22 for an acceptable assembly. The BUSCO results indicated that the assemblies were highly complete, with complete matches ranging from 73–90% of vertebrate orthologs (Table 2). These BUSCO benchmark values are consistent with the most complete reported assessments for transcriptomes from other vertebrate taxa (busco.ezlab.org). Furthermore, our BUSCO values exceed that of the only available reported male reproductive tissue (from a coelacanth: *Latimeria menadoensis* testes), which was 71% complete (Simão et al., 2015). The assembly version with the highest quality in relation to the Transrate metrics was the Transrate optimized Trinity assembly. Specifically, the optimized Transrate score was 0.3492, and the percent coverage of the reference assembly was also highest, with 45% of the mouse database represented. This assembly was highly competitive for completeness, as indicated by the fact that it contained 85% of vertebrate single copy orthologs. However, this assembly had an exorbitantly high number of contigs (657,952 contigs), which is nearly an order of magnitude more contigs than the next best performing assembly: the Transrate optimized TPM > 0.5 filtered assembly (78,424 contigs). In consideration of the dramatically more realistic contig number for the Transrate optimized TPM > 0.5 filtered assembly, and in light of its second best performance for Transrate score (0.3013), reasonable Transrate mouse reference assembly coverage (37%), and sufficiently high BUSCO completeness (73% orthologs found), we chose this assembly as our optimized transcriptome. Therefore, we proceeded with this optimized assembly version as our finalized transcriptome assembly for our analyses.

**Table 1:** Transrate results for the reproductive transcriptome assembly produced by different optimization methods.

Assembly	Transrate score	Optimized score^a^	# Read pairs (fragments)	Contigs (n_seqs)	# Good contigs	% Good contigs
Trinity original	0.1944	0.3492	207,980,214	856,711	657,952	0.77
Filter TPM < 0.5	0.1672	0.3013	207,980,214	147,966	78,424	0.53
Filter TPM < 1.0	0.156	0.2854	207,980,214	80,165	54,140	0.68

**Notes.**

a This is the score of the Transrate optimized assembly in Table 2.

**Table 2:** BUSCO metrics for the reproductive transcriptome assembly produced by different optimization methods.

Assembly	% Complete	% Duplicated	% Fragmented	% Missing
Trinity original	90	49	3.4	5.5
Transrate optimized	85	44	4.3	9.7
Filter TPM < 0.5	85	38	3.0	11
Transrate TPM < 0.5	73	31	3.9	22
Filter TPM < 1.0	80	28	2.8	16
Transrate TPM < 1.0	74	25	3.4	21

### Annotation, transcript abundance and database searches

The reproductive transcriptome assembly annotations were produced by dammit, and they are available on Dryad in a gff3 file format. Furthermore, TransDecoder was used to predict coding regions in the assembly. TransDecoder predicted that 49.5% (38,342) of the transcripts (78,424 total) contained ORFs, of which 63.9% (24,808) had complete ORFs containing a start and stop codon. The predicted protein coding regions generated by TransDecoder are reported in five file types, and they are available on Dryad. Furthermore, the Pfam results yielded 30.7% of transcripts (24,107) matching to the protein family database. In contrast, the LAST search found that 75.9% of transcripts (59,503) matched to the UniRef90 database. We have uploaded the homology search results generated by Pfam and UniRef90 matches onto Dryad. In addition, 1.04% (816) of transcripts matched to the Rfam database for ncRNAs, and these results are posted in Dryad. Of note, 80.1% (62,835) of the transcripts were annotated using one or more of the above described methods (the dammit.gff3 file is posted in Dryad), and it is this final annotated assembly that was used for all subsequent analyses (this annotated transcriptome is available on Dryad).

**Figure 1:**
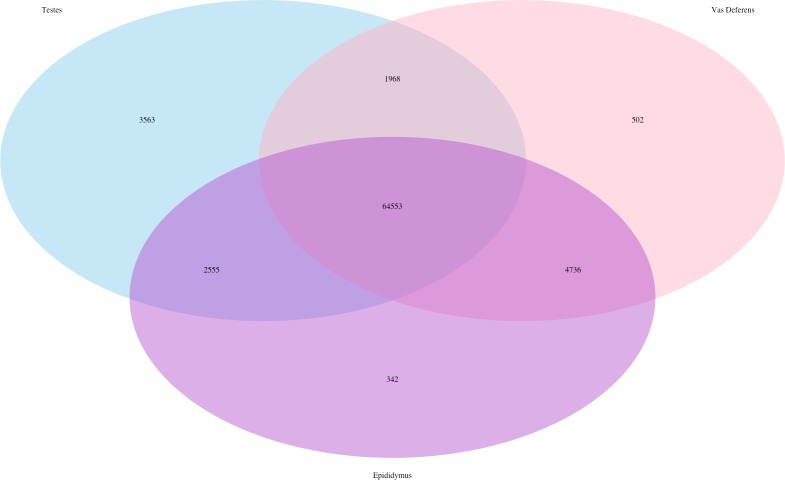
Venn Diagram of transcript expression differences and similarities between the three reproductive tissues for a single male mouse. The total number of transcripts is 78,424.

The Kallisto generated TPM counts of expression (available on Dryad) were utilized to determine which transcripts were ubiquitous and specific to the three tissue types, which we have depicted in a Venn diagram format (Fig. 1). The assembly consisted of 78,424 different transcript IDs, of which 64,553 were shared across all three tissues. The number of unique transcripts were as follows: 3,563 in testes, 342 in epididymis, and 502 in vas deferens. The relatively large number of unique transcripts in the testes is consistent with previous findings which describe the testes as the tissue with the highest number of tissue-enriched genes in the human body (Djureinovic et al., 2014; Uhlén et al., 2015). However, because this expression data was generated by a single individual, we want to emphasize that these results have no statistical power. Rather, we view these numerical comparisons of unique and ubiquitous transcript counts as an exploratory evaluation of potential relationships between tissue types. Such comparisons across tissue types for a single individual of this species previously indicated that the kidney had relatively higher numbers of unique transcripts than testes (MacManes & Eisen, 2014). Future research with multiple individuals will be necessary to statistically evaluate the relative rates of transcript expression between these reproductive tissues, as well as their relationship with other non-reproductive tissue types.

**Figure 2:**
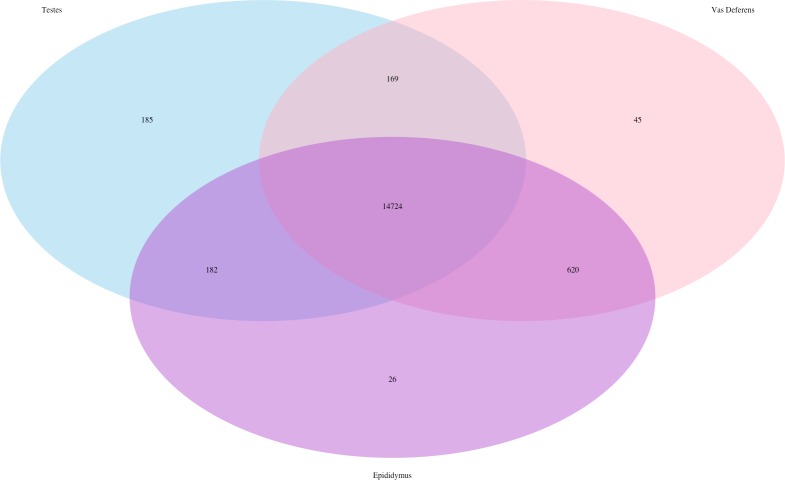
Venn Diagram of transcript matches between the three reproductive tissues to ncRNA sequences in *Mus musculus*. The total number of transcript matches across the tissue types is 15,964.

In addition, we searched for *Mus musculus* ncRNA sequence matches within our assembly. There were 15,964 transcript matches, which correspond to 2,320 unique ncRNA matches, and they are posted on Dryad. The transcript matches by tissue type were found using the Kallisto TPM determinations, and they were as follows, testes: 15,260, epididymis: 15,552, and vas deferens: 15,558. A Venn Diagram depicts unique and shared transcript matches by tissue type (Fig. 2). The majority of transcript matches were ubiquitous to all three tissues (14,724), and there were far fewer tissue specific matches. The testes had more unique transcript matches (185) than the epididymis (26) or the vas deferens (45). These findings are consistent with our results above regarding the relative numbers of total unique transcripts in the assembly by tissue type. However, these counts for relative transcript matches among tissue types were generated with transcripts from a single individual; therefore, the comparative results across tissue type were not statistically evaluated. It is seemingly probable that the diversity of transcripts for regulation should be highest in tissues generating relatively diverse proteins, in this case, the testes, which did have the highest number of unique transcript matches. The role of ncRNA in reproductive tissues throughout multiple developmental stages has recently been reviewed in detail (Hale, Yang & Ross, 2014).  In addition, ncRNAs have been found to be highly abundant in murine testes (Sun, Lin & Wu, 2013). Furthermore, sperm from humans and mice contain a significant number of ncRNAs (Krawetz et al., 2011; Kowano et al., 2012). However, we are unaware of any research investigating the involvement of ncRNA in desert adaptations; therefore, we cannot speculate on particular ncRNA matches within our dataset that may have potential desert adaptive roles.

**Figure 3:**
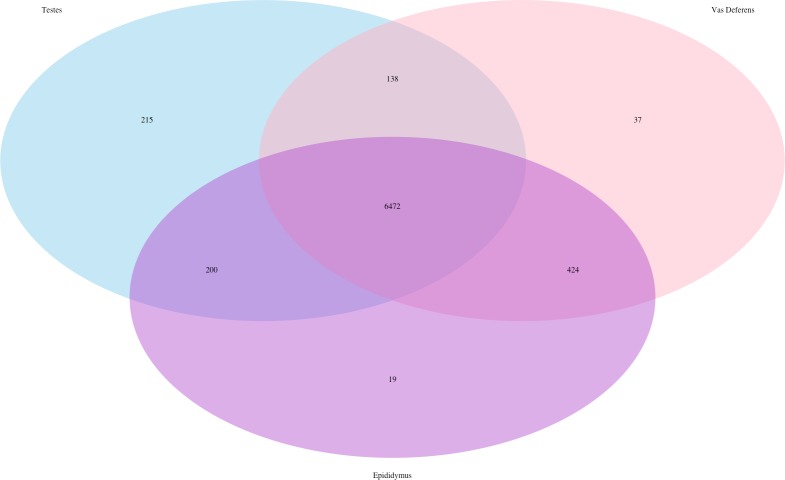
Venn Diagram of transcript matches between the three reproductive tissues to protein sequences in the Transporter Classification Database. The total number of transcript matches across the tissue types is 7,521.

Our search for transporter protein matches within the Transporter Classification Database yielded 7,521 different transcript matches, corresponding to 1,373 unique transporter protein matches, and they are posted on Dryad. The number of transcript matches was highly similar between the tissue types (testes 7,025; epididymis 7,115; vas deferens: 7,071). We generated a Venn Diagram to display the numbers of shared and unique transcript matches to the transporter protein sequences (Fig. 3). Most transcript matches were present in all three tissues (6,472), and there were relatively few unique matches in the three tissue types. However, the testes had the highest number of unique transcript matches (215) relative to the epididymis (19) and the vas deferens (37). These comparative results across tissue types represent exploratory findings for a single mouse, and the count data have not been statistically tested, but these preliminary results should be investigated in future studies. Furthermore, our BLASTx search of this transporter protein database yielded transcript matches for multiple solute carrier proteins. We are particularly interested in solute carrier proteins because previous research has found candidate genes in this protein family for desert adaptations in kidneys of the kangaroo rat (Marra et al., 2012; Marra, Romero & DeWoody, 2014) and the Cactus mouse (MacManes & Eisen, 2014). In addition, we had multiple matches to aquaporins, which are water channels allowing transport across cellular membranes. One transcript matched specifically to Aquaporin 3, a sperm water channel found in mice and humans, which is essential to maintaining sperm cellular integrity in response to the hypotonic environment within the female reproductive tract (Chen et al., 2011).

### Comparative analysis for genes under positive selection

To find evidence for genes undergoing positive selection in the male reproductive transcriptome of *P. eremicus*, we compared this species with two other generalist rodents. We chose *M. musculus* as the non-desert adapted outgroup because this species possesses transcriptomic resources which are exceptional in their annotation and completeness. The widely distributed (Carleton, 1989) habitat generalist deer mouse, *P. maniculatus,* was chosen because it harbors the most complete transcriptomic data available among the *Peromyscus* genus.

There were 3,731 panorthologous groups (single copy orthologs) in our three species comparison. The branch test was successfully implemented for SCOs when all three sequences aligned adequately with PRANK and when codeml produced both *M*1*a* and *M*2*a* output files for the LRT comparison (*n* = 2, 820 in total). The *M*2*a* test indicated that 42 genes were evolving under a model of positive selection in the Cactus mouse (Table 3). Therefore, we investigated whether previous research on either rodent reproductive tissues or on desert specialized rodents documented evidence of positive selection for any of these genes or gene families. Only one of these 42 genes matched an epididymis-specialized secreted gene undergoing positive selection in another rodent (C57/BL6 mice). Namely, Qsox2 is a match for Qscn6l1, a member of the sulfhydryl oxidase/quiescin-6 family, which is purportedly involved in neuroblastoma apoptosis (Dean, Good & Nachman, 2008). However, we cannot speculate regarding the functionality this gene has in male rodent reproductive tissue, or why it appears to be evolving under a model of positive selection in these two studies. Another of our 42 positively selected genes, Lrrc46, may have some similarity to Lrrc50, a gene under positive section in testes of *Peromyscus* (Turner, Chuong & Hoekstra, 2008). Leucine-rich repeat containing (Lrrc) genes have diverse biological roles; therefore, we also will not speculate on any correspondence between these two genes. As expected given our experimental design, there was no concordance between our 42 genes and those found to be under positive selection in a recent study on mouse spermatozoa proteins (Vicens, Luke & Roldan, 2014).

**Figure 4:**
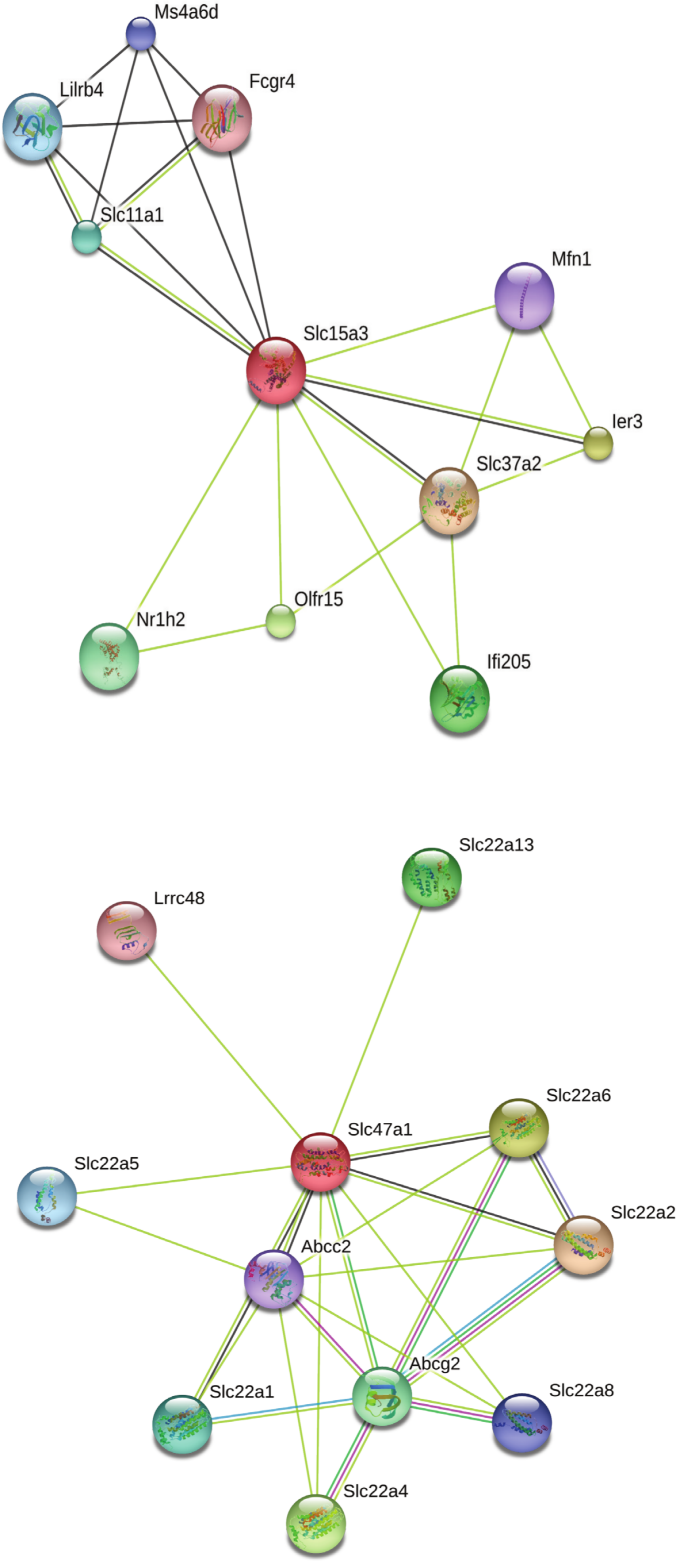
STRING diagrams of protein interactions for two proteins evolving under a model of positive selection in *P. eremicus*: Slc15a3 and Slc47a1.

Our search for gene matches from the current study with other desert rodent research revealed notable similarities. Two solute carrier proteins, Slc15a3 and Slc47a1, were found to be under positive selection in our analysis. This finding bears particular significance because another protein in this family, Slc2a9, shows signatures of positive selection in desert rodent kidney transcriptomes in *Dipodomys spectabilis* and *Chaetodipus baileyi* (Marra, Romero & DeWoody, 2014) and *P. eremicus* (MacManes & Eisen, 2014). Solute carriers are a large family of cell membrane proteins that are responsible for transporting solutes (reviewed in Hediger et al., 2004; Hediger et al., 2013; César-Razquin et al., 2015). Furthermore, Marra, Romero & DeWoody (2014), hypothesize that solute carriers are critical for osmoregulation in desert rodents, and they assert that these genes may be under evolutionary pressure in such rodents. In response to the potential relevance of the two solute carriers under positive selection in our study to desert rodent osmoregulation, we generated STRING (Snel et al., 2000; Szklarczyk et al., 2015) diagrams for their protein-protein interactions (Fig. 4). These diagrams demonstrate multiple connections to other solute carriers for both proteins, thereby suggesting their potential functional roles.

**Table 3:** The 42 genes that reached statistical significance (*p* < 0.05) after correcting for multiple hypothesis testing for the *M2a* branch-site test for positive selection in PAML in the male Cactus mouse reproductive transcriptome.

Orthogroup ID	*p*-value	BLASTn description
OG0010592	1.18E–10	Q6 sulfhydryl oxidase 2 (Qsox2)
OG0010774	1.04E–03	Adenylate kinase 6 (Ak6)
OG0010833	4.90E–05	Plakophilin 2 (Pkp2)
OG0011177	3.15E–03	1-acylglycerol-3-phosphate O-acyltransferase 2 (Agpat2)
OG0011272	1.06E–04	cDNA sequence BC089491
OG0011374	2.83E–03	Hepatoma derived growth factor-like 1 (Hdgfl1)
OG0011384	2.05E–03	Chitinase, acidic 1 (Chia1)
OG0011551	7.05E–04	Zinc finger protein 770 (Zfp770)
OG0011784	1.63E–04	Zinc finger, DHHC domain containing 19 (Zdhhc19)
OG0011914	1.33E–09	Fumarylacetoacetate hydrolase domain containing 2A (Fahd2a)
OG0012115	0.00E+00	NUT midline carcinoma, family member 1 (Nutm1)
OG0012232	2.06E–03	Leucine rich repeat neuronal 2 (Lrrn2)
OG0012396	1.19E–06	Leucine rich repeat containing 46 (Lrrc46)
OG0012449	2.79E–04	F-box and leucine-rich repeat protein 14 (Fbxl14)
OG0012511	3.27E–08	SMAD family member 6 (Smad6)
OG0012690	0.00E+00	Solute carrier family 47, member 1 (Slc47a1)
OG0012869	4.59E–02	Bromodomain and WD repeat domain containing 3 (Brwd3)
OG0013171	5.47E–06	ArfGAP with SH3 domain, ankyrin repeat and PH domain 3 (Asap3)
OG0013288	6.09E–03	Phosphodiesterase 3B, cGMP-inhibited (Pde3b)
OG0013304	3.42E–02	Persephin (Pspn)
OG0013342	4.11E–02	Mitochondrial methionyl-tRNA formyltransferase (Mtfmt)
OG0013590	1.77E–02	Matrix metallopeptidase 15 (Mmp15)
OG0013771	1.77E–02	Neuronal pentraxin 2 (Nptx2)
OG0013841	4.34E–03	Transformation related protein 63 regulated like (Tprgl)
OG0014000	1.25E–02	Alkaline phosphatase, liver/bone/kidney (Alpl)
OG0014048	0.00E+00	BPI fold containing family A, member 5 (Bpifa5)
OG0014062	1.42E–02	SPARC related modular calcium binding 2 (Smoc2)
OG0014193	1.05E–06	Carbonic anhydrase 11 (Car11)
OG0014286	9.65E–03	cDNA 4930550C14 gene
OG0014309	7.40E–03	Chymotrypsin-like elastase family, member 1 (Cela1)
OG0014333	2.11E–02	Minichromosome maintenance 9 homologous recombination repair factor (Mcm9)
OG0014414	1.60E–04	Growth arrest specific 6 (Gas6)
OG0014634	2.62E–03	Glutathione peroxidase 2 (Gpx2)
OG0014827	0.00E+00	Secretory carrier membrane protein 5 (Scamp5)
OG0014913	4.34E–04	STIP1 homology and U-Box containing protein 1 (Stub1)
OG0015025	3.23E–08	Pancreatic lipase-related protein 2 (Pnliprp2)
OG0015310	1.06E–03	Claspin (Clspn)
OG0015466	7.62E–05	Excision repair cross-complementing rodent repair deficiency complementation group 6 like (Ercc6l)
OG0015627	4.18E–03	Solute carrier family 15, member 3 (Slc15a3)
OG0015662	1.52E–03	Epithelial membrane protein 2 (Emp2)
OG0015704	3.96E–03	Cubilin (intrinsic factor-cobalamin receptor) (Cubn)
OG0015713	4.97E–07	Suppressor APC domain containing 1 (Sapcd1)

This analysis of genes undergoing positive selection in *P. eremicus* relative to *P. maniculatus* and *M. musculus* provides candidate genes for desert specialization in the Cactus mouse which can be the target of future studies focused on ascertaining which genes may be functionally responsible for our hypothesized male reproductive desert adaptation. However, desert specialization is not the sole difference between *P. eremicus* and *P. maniculatus*, much less between *P. eremicus* and *M. musculus*. *P. maniculatus* is highly promiscuous, while *P. eremicus* is relatively socially monogamous (Wolff, 1989). Indeed, *P. maniculatus* has been the subject of considerable sperm competition research (Dewsbury, 1988; Fisher & Hoekstra, 2010). Differences in reproductive systems, and even potentially in sperm aggregation of *P. maniculatus* (Fisher & Hoekstra, 2010), may manifest themselves as evidence of selection patterns between these two *Peromyscus* species. Therefore, we are not proposing that the 42 genes we found to be under positive selection in the male Cactus mouse are functionally responsible for adaptive desert physiology. Rather, we are proposing that they are interesting candidate genes for future studies investigating the genetic underpinnings of physiological desert adaptations, including our hypothesized male reproductive adaptation, on a functional level. Several of these genes, specifically those in the solute carrier family, seem particularly promising for such work because they are undergoing rapid evolution in multiple desert rodent species.

## Conclusions

Although researchers have determined that renal adaptations are responsible for mitigating water loss in kangaroo rats via the genitourinary tract (Schmidt-Nielsen et al., 1948; Schmidt-Nielsen & Schmidt-Nielsen, 1952; Vimtrup & Schmidt-Nielsen, 1952; Urity et al., 2012), we present the novel hypothesis that there may also be male reproductive adaptations to arid environments that allow desert specialists like the Cactus mouse to conserve water during reproduction. Previous efforts to elucidate the genomic basis of desert adaptations have described candidate genes for adaptive renal physiology in some desert specialized rodents (Marra et al., 2012; Marra, Romero & DeWoody, 2014), including the Cactus mouse (MacManes & Eisen, 2014). In light of these findings, we propose that if the male Cactus mouse possesses an adaptive reproductive phenotype to mitigate water loss via seminal fluids in response to limited-water availability, such an adaptation will be detectable through transcriptomic analyses. The current study generates and characterizes a transcriptome for male reproductive tissues from the Cactus mouse as an initial step towards future efforts to explore this hypothesized reproductive adaptation. This study describes a composite transcriptome from three male reproductive tissues in the desert specialist *Peromyscus eremicus*. Our analyses include quality and completeness assessments of this reproductive assembly, which we generated using reads from testes, epididymis, and vas deferens of a male Cactus mouse. We generate annotations and search relevant databases for ncRNAs and transporter protein sequences. We also describe the degree of ubiquity between transcripts among the three tissues as well as identify transcripts unique to those tissues utilizing preliminary (based on a single individual) transcript differences between tissue types. Furthermore, we find genes evolving under a model of positive selection in the *P. eremicus* male reproductive transcriptome relative to *P. maniculatus* and *M. musculus* in order to generate a list of candidate genes for future investigations in desert adaption genetics. Our future research will investigate the hypothesized male reproductive physiological adaptation to water limitation in Cactus mouse through a differential gene expression study, and the characterization of this reproductive transcriptome will form the foundation of studies along this vein. Moreover, this research contributes transcriptomic materials to a larger body of work in the expanding field of adaptation genetics, which benefits tremendously from enhanced opportunities for comparative analyses.

## Dryad Data File List

Final Annotated Reproductive Tissue Transcriptome: *reproductive.annotated.fasta* (127 MB)

Transdecoder (Five Files):

*transdecoder.gff3* (49 MB)
*transdecoder.pep* (27 MB)
*transdecoder.cds* (62 MB)
*transdecoder.mRNA* (172 MB)
*transdecoder.bed* (10 MB)

Pfam Annotation: *reproductive.pfam.gff3* (32 MB)

Rfam Annotation: *reproductive.rfam.gff3* (191 KB)

Dammit Annotation: *reproductive.dammit.gff3* (98 MB)

UniRef90 Annotation: *reproductive.uniref.gff3* (9.5 MB)

Kallisto Results for Annotated Transcriptome (Three Files):

*kallisto.testes.tsv* (3.4 MB)
*kallisto.epi.tsv* (3.4 MB)
*kallisto.vas.tsv* (3.4 MB)

ncRNA Database Matches (three files):

*epi.tpm.plus.ncRNA.txt* (4.3 MB)
*vas.tpm.plus.ncRNA.txt* (4.3 MB)
*testes.tpm.plus.ncRNA.txt* (4.3 MB)

tcdb Database Matches (three files):

*epi.tpm.plus.tcdb.txt* (946 KB)
*vas.tpm.plus.tcdb.txt* (946 KB)
*testes.tpm.plus.tcdb.txt* (946 KB)

Comparative analysis for genes under positive selection (three files):

PAMLresults.txt (354 KB)
significant_orthogroups.txt (6 KB)
42SeqsM2aPosSel.txt (89 KB).

## Additional Information and Declarations

### DNA Deposition

The following information was supplied regarding the deposition of DNA sequences: European Nucleotide Archive (PRJEB13364, ID: 318672).

### Data Availability

The following information was supplied regarding data availability: Dryad DOI: 10.5061/dryad.01c3t; Github:
https://github.com/macmanes-lab/peer_reproductive-transcriptome.
